# Energy drink consumption among 12- to 17-year-olds in Germany – Results of EsKiMo II

**DOI:** 10.25646/6400

**Published:** 2020-03-04

**Authors:** Franziska Lehmann, Katerina Vesela, Marjolein Haftenberger, Clarissa Lage Barbosa, Gert B. M. Mensink

**Affiliations:** 1 Robert Koch Institute, Berlin Department of Epidemiology and Health Monitoring; 2 Formerly Robert Koch Institute, Berlin Department of Epidemiology and Health Monitoring

**Keywords:** ENERGY DRINKS, CAFFEINE, ADOLESCENTS, ESKIMO II, KIGGS WAVE 2, HEALTH MONITORING

## Abstract

Energy drinks are soft drinks that usually contain a large content of caffeine and sugar. Excessive caffeine intake can lead to side effects such as nausea and anxiety. Up to three milligrams of caffeine per kilogram of body weight per day is considered safe for children and young people. The second Eating study as a KiGGS Module (EsKiMo II, 2015–2017) collected nationwide representative data about children’s and adolescents’ dietary behaviour. To collect food intake data from 12- to 17-year-olds (n=1,353), a dietary history interview was used. 8.9% of the girls and boys stated that they had consumed energy drinks during the four-week reference period, with nearly a quarter of these individuals (n=99) exceeding the limit of safe caffeine intake solely through their consumption of energy drinks. This corresponds to 2.2% of the 12- to 17-year-olds in Germany. In addition to a general warning about the high levels of sugar present in sugary drinks, awareness also needs to be raised among young people about the dangers of excessive caffeine intake resulting from the consumption of energy drinks. Regulations governing sales and advertising should also be considered.

## Introduction

In recent years, sales of energy drinks have risen sharply throughout the world [1]. These drinks are particularly popular among younger people. Manufacturers often aim their drinks at this target group and do so under the premise of improved concentration and strengthened performance [2, 3]. Energy drinks contain high levels of caffeine. Caffeine occurs naturally in plant constituents, and is traditionally consumed in the form of coffee, tea, soft drinks and processed cocoa powder [4]. Energy drinks usually contain 32 milligrams of caffeine per 100 millilitres, which is twice the level found in commercially available cola drinks. However, energy drinks contain other substances such as taurine, glucuronolactone and inositol, which can also have physiological effects, [5] and they usually contain high levels of sugar.

However, it is the high caffeine content found in commercially-available energy drinks that has recently led researchers to focus increasingly on these products. For example, in 2015, the European Food Safety Authority (EFSA) published a report on the safety of caffeine. The report includes guidelines for caffeine intake from all dietary sources for the general healthy population as well as for subgroups such as children and adolescents. The EFSA recommends that young people should not consume more than three milligrams of caffeine per kilogram of body weight in one day or in a single dose [6]. Excessive caffeine intake can lead to nausea, headache, anxiety and cardiovascular problems. This is particularly the case with people who are sensitive to caffeine [4].

**KiGGS Wave 2**

Second follow-up to the German Health Interview and Examination Survey for Children and Adolescents

**Data owner:** Robert Koch Institute

**Aim:** Providing reliable information on health status, health-related behaviour, living conditions, protective and risk factors, and health care among children, adolescents and young adults living in Germany, with the possibility of trend and longitudinal analyses

**Study design:** Combined cross-sectional and cohort study

**Cross-sectional study in KiGGS Wave 2**

**Age range:** 0–17 years

**Population:** Children and adolescents with permanent residence in Germany

**Sampling:** Samples from official residency registries - randomly selected children and adolescents from the 167 cities and municipalities covered by the KiGGS baseline study

**Sample size:** 15,023 participants

**KiGGS cohort study in KiGGS Wave 2**

**Age range:** 10–31 years

**Sampling:** Re-invitation of everyone who took part in the KiGGS baseline study and who was willing to participate in a follow-up

**Sample size:** 10,853 participants

**KiGGS survey waves**

- KiGGS baseline study (2003–2006), examination and interview survey
- KiGGS Wave 1 (2009–2012), interview survey
- KiGGS Wave 2 (2014–2017), examination and interview survey

More information is available at www.kiggs-studie.de/english

Sugary drinks are not recommended from a nutritional standpoint as they can increase the risk of overweight and dental caries [7]. As adolescents are still in the development and growth phase, they have a particular need for an optimal supply of energy and nutrients. Despite the decline in the consumption of sugary drinks observed during the second follow-up of the German Health Interview and Examination Survey for Children and Adolescents (KiGGS Wave 2, 2014–2017), the amount of sugary drinks currently being consumed is still considered to be too high [8].

In the following, data from the second Eating study as a KiGGS Module (EsKiMo II) are used to estimate the proportion of 12- to 17-year-olds that consume energy drinks in Germany. Data from EsKiMo II are also used to show the percentage of girls and boys whose caffeine intake exceeds safe levels. The findings are described in relation to sex, age and family socioeconomic status (SES).

## Indicator

EsKiMo II (2015–2017) was implemented within the framework of KiGGS Wave 2 (2014–2017). KiGGS is part of the health monitoring system at the Robert Koch Institute and includes repeated cross-sectional surveys of children and adolescents aged 0 to 17 that are representative for Germany (KiGGS cross-sectional study). The KiGGS baseline study (2003–2006) was conducted as an examination and interview survey, KiGGS Wave 1 (2009–2012) as a telephone-based interview survey and KiGGS Wave 2 (2014–2017) as an examination and interview survey. The concept and design of KiGGS Wave 2 have been described in detail elsewhere [9, 10].

As part of the EsKiMo study, nationwide representative data were collected on the nutritional behaviour of 6- to 17-year-old children and adolescents in Germany. Data on the dietary intake of 12- to 17-year-olds was gathered for EsKiMo II with a computer-assisted, modified dietary history interview using the Dietary Interview Software for Health Examination Studies (DISHES) [11]. Trained nutritionists conducted face-to-face interviews with the participants about their usual eating habits at mealtimes. The questions covered the previous four weeks. Data were also collected on energy drink intake, including the quantity of drinks that the participants consumed. Data were collected on portion sizes by demonstrating standard plate and cup sizes, using a picture book [12, 13] or by providing information about commercially-available portion sizes. The DISHES software package includes Version 3.02 of the German Nutrient Database (BLS) [14] and additional data on specific foods. Detailed information about the methodology and the study design implemented for EsKiMo II is available elsewhere [15, 16].

The following analyses are based on data from 1,353 children and adolescents (727 girls, 626 boys) aged 12 to 17. The proportion of participants who stated that they had consumed energy drinks at least once in the four-week period is stratified by sex, age and socioeconomic status (SES) [17].

Caffeine intake was evaluated using the EFSA’s recommended safe levels of caffeine for children and adolescents [6]. Caffeine intake from energy drinks was calculated using data on the daily intake of energy drinks (grams per day) and the level of caffeine that these drinks contain (milligrams per 100 grams). A caffeine database was composed within EsKiMo II to document the caffeine levels present in various foodstuffs. The figures on caffeine contained in the database are the result of a literature review undertaken by the authors, as well as figures provided by the Federal Office of Consumer Protection and Food Safety (BVL) and the Chemical and Veterinary Examination Office Karlsruhe (CVUA). Information on body weight is based on self-reported data collected during the DISHES interviews.

**EsKiMo II**

Second Wave of the Eating study as a KiGGS Module, 2015–2017

**Acronym: EsKiMo – E**ating study as a **K**iGGS **M**odule

**Implementation:** Robert Koch Institute

**Aim:** Providing an up-to-date representative overview of the dietary habits of children and adolescents aged 6 to 17 in Germany.

**Study design:** Cross-sectional study based on a modified diet history interview and food records

**Population:** Children and adolescents with permanent residence in Germany

**Sampling:** EsKiMo II participants are randomly selected from the cross-sectional sample of KiGGS Wave 2 (registry office sample). Being invited to EsKiMo II requires participation in KiGGS Wave 2.

**Age range:** 6 to 17 years

**Sample size:** 2,644 participants

**Survey period:** June 2015 – September 2017

More information in German is available at www.rki.de/eskimo

The calculations were carried out using a weighting factor that corrects deviations within the EsKiMo II sample from the population structure with regard to regional structure (rural area/urban area), age (in years), sex, federal state (as of 31 December 2015), German citizenship (as of 31 December 2014), parental education level (Microcensus 2013 [18]) and differences in survey participation according to seasonality, family socioeconomic status and a child’s school type.

The results are presented as prevalences (frequencies) with 95% confidence intervals (95% CI). Prevalences are estimates, the precision of which can be assessed through the use of confidence intervals; wide confidence intervals indicate greater statistical uncertainty in the results. A statistically significant difference between groups is assumed when the corresponding p-value is less than 0.05, after taking into account weighting and the survey design.

## Results and discussion

8.9% of the young people state that they have consumed energy drinks over the last four weeks. Consumption of energy drinks among both sexes increases with age: almost twice as many young people aged between 16 and 17 consume energy drinks as those aged between 12 and 13. However, this difference is only statistically significant for boys. Almost the same percentage of girls (9.7%) as boys (8.7%) consume energy drinks. 12- to 17-year-olds from families with a low SES have an almost three times more frequent intake of energy drinks than those from families with a high SES (15.8% vs 5.4%). Nevertheless, this difference is not statistically significant (Table 1).

The use of different survey instruments, reference periods and age groups complicates attempts to compare these results with findings from previous international studies. In a 2012 survey of 16 European Union member states, which was commissioned by the EFSA, around 60% of 1,068 children and adolescents aged between 10 and 18 surveyed in Germany stated that they had consumed energy drinks in the year leading up to the survey [1]. Furthermore, the Prevention Radar (2019) of the health insurance company DAK-Gesundheit found that 13% of school pupils in Germany had consumed energy drinks before and during school in the last 30 days. However, the majority of pupils drank energy drinks less than once a month [19]. As these results imply that energy drinks are consumed on an irregular basis, the four-week survey period in EsKiMo II may have led the study to slightly underestimate the actual figures.

Energy drinks account for 4.3% of total caffeine intake (from all types of caffeinated drinks) by young people in Germany. Analyses of estimates of caffeine intake using data from the Dortmund Nutritional and Anthropometric Longitudinally Designed Study (DONALD) also show that energy drinks only account for a small proportion of total caffeine intake from all types of caffeine-containing beverages [20]. Slightly under a quarter (24.3%) of energy drink consumers (n=99) exceed the EFSA’s safe caffeine intake of three milligrams per kilogram of body weight per day or in a single dose. This corresponds to 2.2% of the young people in the studied age group. However, since these results are based on self-reported data about dietary intake, and it is possible that the participants provided socially desirable responses, the results may underestimate the actual level of caffeine intake.

A moderate dietary intake of energy drinks and other caffeinated drinks is considered harmless [6]. However, the results from EsKiMo II show that one in four energy drink consumers (and this applies to girls and boys) exceed the safe intake of caffeine through their consumption of energy drinks alone. The German Federal Institute for Risk Assessment’s survey of people with a particularly high intake of energy drinks also indicates that on some occasions these individuals consume excessively large quantities amounting to one litre or more [2]. Bans on the sale of energy drinks (particularly in schools) and restrictions on marketing aimed at young people, therefore, should be examined at the national level in order to minimise the risk of the side effects linked to excessive caffeine intake. Other countries have already put regulatory measures in place in this regard [3]. Lowering the maximum permissible level of caffeine in energy drinks represents a further possibility, particularly as manufacturers tend to ensure that their products contain the highest permissible level of caffeine.

Energy drinks are sugary drinks. In December 2018, the German Federal Ministry of Food and Agriculture published a National Reduction and Innovation Strategy, which also included the sugar found in convenience products [21]. Further measures will probably have to be implemented in order to significantly reduce the levels of sugar that people consume. For example, a higher tax on drinks that contain large amounts of sugar is currently discussed. Evaluations from other countries show that sugar taxes help to reduce the sale of sugary soft drinks [22].

## Key statements

- Almost 9% of 12- to 17-year-olds consumed energy drinks during a four-week period.
- Nearly a quarter of energy drink consumers exceeded safe levels of caffeine intake.
- No differences in the consumption of energy drinks were identified between the sexes.
- Energy drinks were consumed about twice as often by 16- to 17-year-olds as by 12- to 13-year-olds.

## Figures and Tables

**Table 1:** Prevalence of energy drink consumption by sex, age and socioeconomic status (n=727 girls, n=626 boys) Source: EsKiMo II (2015–2017)

%		(95% CI)	%		(95% CI)
Girls (total)	9.1	(5.8–12.3)	Boys (total)	8.7	(5.9–11.4)
**Age group** 12–13 years 14–15 years 16–17 years	7.7 7.1 12.2	(1.8–13.5) (2.0–12.3) (6.1–18.2)	**Age group** 12–13 years 14–15 years 16–17 years	5.3 7.0 13.3	(2.2–8.3) (3.3–10.7) (6.7–19.9)
**Socioeconomic status** Low Medium High	19.4 6.2 6.6	(8.0–30.8) (3.4–9.0) (0.0–15.1)	**Socioeconomic status** Low Medium High	11.0 9.8 4.6	(0.7–21.2) (6.3–13.3) (0.8–8.3)
**Total (girls and boys)**	8.9	(6.7–11.0)	**Total (girls and boys)**	8.9	(6.7–11.0)

CI = confidence interval
